# Lung Cancer and Exposure to Nitrogen Dioxide and Traffic: A Systematic Review and Meta-Analysis

**DOI:** 10.1289/ehp.1408882

**Published:** 2015-04-14

**Authors:** Ghassan B. Hamra, Francine Laden, Aaron J. Cohen, Ole Raaschou-Nielsen, Michael Brauer, Dana Loomis

**Affiliations:** 1 Department of Environmental and Occupational Health, Drexel University School of Public Health, Philadelphia, Pennsylvania, USA; 2 Channing Division of Network Medicine, Department of Medicine, Brigham and Women’s Hospital and Harvard Medical School, Boston, Massachusetts, USA; 3 Department of Environmental Health, and; 4 Department of Epidemiology, Harvard School of Public Health, Boston, Massachusetts, USA; 5 Health Effects Institute, Boston, Massachusetts, USA; 6 Danish Cancer Society Research Center, Copenhagen, Denmark; 7 School of Population and Public Health, University of British Columbia, Vancouver, British Columbia, Canada; 8 International Agency for Research on Cancer, Lyon, France

## Abstract

**Background and objective:**

Exposure to traffic-related air pollutants is an important public health issue. Here, we present a systematic review and meta-analysis of research examining the relationship of measures of nitrogen oxides (NO_x_) and of various measures of traffic-related air pollution exposure with lung cancer.

**Methods:**

We conducted random-effects meta-analyses of studies examining exposure to nitrogen dioxide (NO_2_) and NO_x_ and its association with lung cancer. We identified 20 studies that met inclusion criteria and provided information necessary to estimate the change in lung cancer per 10-μg/m^3^ increase in exposure to measured NO_2_. Further, we qualitatively assessed the evidence of association between distance to roadways and traffic volume associated with lung cancer.

**Results:**

The meta-estimate for the change in lung cancer associated with a 10-μg/m^3^ increase in exposure to NO_2_ was 4% (95% CI: 1%, 8%). The meta-estimate for change in lung cancer associated with a 10-μg/m^3^ increase in NO_x_ was similar and slightly more precise, 3% (95% CI: 1%, 5%). The NO_2_ meta-estimate was robust to different confounding adjustment sets as well as the exposure assessment techniques used. Trim-and-fill analyses suggest that if publication bias exists, the overall meta-estimate is biased away from the null. Forest plots for measures of traffic volume and distance to roadways largely suggest a modest increase in lung cancer risk.

**Conclusion:**

We found consistent evidence of a relationship between NO_2_, as a proxy for traffic-sourced air pollution exposure, with lung cancer. Studies of lung cancer related to residential proximity to roadways and NO_x_ also suggest increased risk, which may be attributable partly to air pollution exposure. The International Agency for Research on Cancer recently classified outdoor air pollution and particulate matter as carcinogenic (Group 1). These meta-analyses support this conclusion, drawing particular attention to traffic-sourced air pollution.

**Citation:**

Hamra GB, Laden F, Cohen AJ, Raaschou-Nielsen O, Brauer M, Loomis D. 2015. Lung cancer and exposure to nitrogen dioxide and traffic: a systematic review and meta-analysis. Environ Health Perspect 123:1107–1112; http://dx.doi.org/10.1289/ehp.1408882

## Introduction

Exposure to air pollution from high-density urban traffic is an important public health issue. Traffic-related air pollutants are associated with acute health outcomes as well as chronic respiratory and cardiovascular outcomes [Brunekreef et al. 1997; Health Effects Institute (HEI) 2010; Hoek et al. 2002; Tonne et al. 2007]. Associations of traffic-related air pollution with allergies, asthma, and respiratory infections have also been demonstrated in children (Brauer et al. 2002) and in both developed and developing nations (Brunekreef et al. 2009). Evaluation of carcinogenicity of traffic-based pollutants is complicated by the fact that the diseases are rare and often develop after a long latency period. Thus, multiple large, long-term observational studies are required to demonstrate evidence of carcinogenicity from traffic-related air pollution. A recent systematic review and meta-analysis evaluated the evidence from epidemiology of lung cancer associated with particulate matter exposure (Hamra et al. 2014); importantly, road traffic is an important contributor to urban particulate matter. The evidence supported the recent classification of ambient air pollution and particulate matter as a Group 1 carcinogen by the International Agency for Research on Cancer (IARC).

Here, we present a systematic review and meta-analysis of research examining the relationship of measures of nitrogen oxides [measured as nitrogen dioxide (NO_2_) or nitrogen oxides (NO_x_)] and of various measures of traffic exposure and lung cancer. In urban settings, NO_2_ is often used as a marker for traffic-based air pollution, which is a complex mixture of many diverse carcinogens, such as volatile organic compounds, metals, and carbonyls (Brook et al. 2007; Valavanidis et al. 2008). We conducted analyses of studies focused on NO_x_ and NO_2_; the latter is a component of the former, but both have been used as markers of traffic exposure in epidemiological research. We also evaluated studies that used distance from roadways or volume of traffic on nearby roadways as indicators of exposure to traffic-related air pollution (Brook et al. 2007).

## Methods

*Literature search*. We began with a systematic review of the PubMed database using the following search criteria: (traffic[Title/Abstract] OR Nitrogen dioxide[Title/Abstract] OR NO_2_[Title/Abstract] OR NO_x_[Title/Abstract] OR Nitrogen oxide[Title/Abstract]) AND (lung cancer[Title/Abstract]). Studies were further required to be human-based epidemiologic studies written in English. A previous, similar search conducted by Hamra et al. (2014) did not retrieve relevant studies in non-English languages; therefore, we do not believe the language restriction led to exclusion of informative studies. This search was conducted in January 2014, and yielded 179 records. Abstracts from each paper were then reviewed for relevance to the topic of traffic or nitrogen oxide exposure and lung cancer. This review led to 30 studies for initial consideration.

We required abstracts to explicitly provide a quantitative value for change in lung cancer incidence or mortality associated with exposure to nitrogen oxides or other measure of traffic-related air pollution or to suggest that such an estimate was provided in the text. Of the initial 30 abstracts examined, 26 provided this information. Further, we considered only studies that used a case–control or cohort design; thus, ecological studies were not included. Finally, where there were multiple studies that considered the same, or highly overlapping, cohorts, we chose the study that had the longest follow-up time and/or the greater number of lung cancer events.

We abstracted estimates of lung cancer associated with three exposure metrics: NO_2_, NO_x_ (NO + NO_2_), and volume of traffic and distance to nearest traffic source. Further, we abstracted information on confounders for which the authors adjusted in their analyses. All studies adjusted for age and sex.

*Statistical analyses*. For consistency, all study estimates were converted to represent the change in lung cancer incidence or mortality per 10-μg/m^3^ exposure to NO_2_ or NO_x_. All studies that considered NO_x_ used units of micrograms per cubic meter. Studies of NO_2_ measured exposure in units of micrograms per cubic meter or parts per billion. For studies measuring NO_2_ in parts per billion, we used a conversion factor of 1 ppb = 1.88 μg/m^3^, which is based on ambient pressure of 1 atmosphere and a temperature of 25°C (Vrijheid et al. 2011). We abstracted the effect estimates that were believed to most effectively adjust for confounding (i.e., the least biased effect estimates), which were largely the “main models” discussed by the authors. There did not appear to be issues of overadjustment or potential adjustment for causal intermediates or colliders in any studies. Most studies used a single-pollutant model; when authors used both a single- and a multi-pollutant model, we chose estimates from the former.

We used random-effects estimation for our meta-analysis. Unlike fixed-effects estimation, random-effects methods include an estimate of the percent of the total variance attributable to between study inconsistency, referred to as the *I*^2^ value (DerSimonian and Laird 1986; Higgins and Green 2008; Higgins et al. 2003). One study (Abbey et al. 1999) presented estimates by sex; these estimates were combined with fixed-effects estimation and then included in analyses of the overall meta-estimates. This assumes that the stratum-specific estimates are collapsible across strata of sex.

Forest and funnel plots were created to provide a visual representation of the distribution of study-specific effect estimates. In addition, we conducted trim-and-fill analyses, which, in the presence of funnel plot asymmetry, recalculate the meta-estimate based on hypothetical, unobserved studies that would have been necessary to create a symmetrical funnel plot (Duval and Tweedie 2000; Higgins and Green 2008). Finally, we conducted influence analyses, where the meta-estimate is recalculated excluding one study at a time to test if the overall meta-estimate is robust to exclusion of any single study. We did not include formal assessments of study quality. Analyses were conducted using STATA software (version 12.1; StataCorp).

## Results

*Studies included*. We identified 20 studies that met our initial inclusion criteria through PubMed (http://www.ncbi.nlm.nih.gov/pubmed) and three additional studies through discussion among co-authors or by searching article references (Cao et al. 2011; Hart et al. 2011; Puett et al. 2014). Six of the 23 studies had at least partially overlapping populations; of these, the three judged to be less informative were excluded (Beeson et al. 1998; Jerrett et al. 2013; Vineis et al. 2006). More informative studies are those that have either better information on estimated individual exposure or a greater number of observed lung cancers. Of particular note, although the exposure assessment for an updated American Cancer Society Cancer Prevention II Study was more accurate (Jerrett et al. 2013), we considered a slightly older study that benefits from a much larger sample size (Krewski et al. 2009). The final data set included 20 studies that met all required criteria for inclusion in our meta-analyses (Abbey et al. 1999; Beelen et al. 2008; Cao et al. 2011; Carey et al. 2013; Cesaroni et al. 2013; Filleul et al. 2005; Hart et al. 2011; Heinrich et al. 2013; Hystad et al. 2013; Katanoda et al. 2011; Krewski et al. 2009; Lipsett et al. 2011; Nafstad et al. 2003; Nyberg et al. 2000; Puett et al. 2014; Raaschou-Nielsen et al. 2010, 2011, 2013; Villeneuve et al. 2014; Yorifuji et al. 2013). Table 1 summarizes the 20 studies included in our analyses, and reports mean (± SD) of annual, individual exposure to NO_2_ or NO_x_ in units of micrograms per cubic meter or parts per billion, depending on what the authors originally reported. Of these studies, 15 and 4 provide estimates of the change in lung cancer incidence or mortality associated with NO_2_ and NO_x_, respectively; Puett et al. (2014) provide information only for distance from roadways. Because the ratio of NO_2_ to NO_x_ is complex and location-specific, and depends on the time-varying levels of atmospheric oxides, there is no simple conversion factor to estimate the percent of NO_x_ represented by NO_2_. We therefore summarize meta-estimates from studies of NO_x_ separately.

**Table 1:** Description of cohorts included.

Region	Study ID^*a*^	Reference	No. of events	Total population	Study period	Exposure assessment method^*b*^	Mean (± SD) annual, individual exposure^*c*^	Study
North America
California, USA	1	Abbey et al. 1999	29 (mortality)	5,652	1977–1992	Fixed-site monitor		Adventist Health Study on Smog (AHSMOG)
USA	2	Krewski et al. 2009	16,615 (mortality)	499,968	1982–2000	Fixed-site monitor	27.9 (85.25) ppb	American Cancer Society Cancer Prevention Study II
USA	3	Hart et al. 2011	800 (mortality)	53,814	1985–2000	Spatiotemporal model	14.2 (7.1) ppb	Trucking Industry Particle Study (TrIPS)
California, USA	4	Lipsett et al. 2011	67 (mortality)	12,366	1997–2005	Inverse distance weighting	33.6 (9.6) ppb	California Teachers Study (women only)
Canada	5	Hystad et al. 2013^*d,e*^	2,390 (incidence)	5,897	1994–1997	Spatiotemporal model	15.4 (9.0) ppb	National Enhanced Cancer Surveillance System case–control study
Toronto, Canada	6	Villeneuve et al. 2014^*d*^	327 (incidence)	752	1997–2002	Land use regression	22.3 (NR) ppb	Study of four tertiary care hospitals in Toronto
USA	NA	Puett et al. 2014^*e*^	1,648 (incidence)	97,865	1998-2010	Spatiotemporal model	NA	Nurses’ Health Study
Europe
Stockholm, Sweden	7	Nyberg et al. 2000^*d*^	1,042 (incidence)	3,406	1985–1990	Air dispersion	NR	Residents of Stockholm County (1950–1990)
Oslo, Norway	NA	Nafstad et al. 2003^*a,f*^	418 (incidence)	16,209	1972–1998	Air dispersion	10.7 (NR) μg/m^3^	Norwegian Cancer Registry (Oslo, men only)
France	8	Filleul et al. 2005	178 (mortality)	14,284	1974–1998	Fixed-site monitor	NR	Air pollution and chronic respiratory diseases
Netherlands	9	Beelen et al. 2008^*e*^	1,940 (incidence)	120,852	1986–1997	Land use regression	36.9 (NR) μg/m^3^	Netherlands Cohort Study of Diet and Cancer.
United Kingdom	10	Carey et al. 2013	5,241 (mortality)	830,429	2003–2007	Air dispersion	22.5 (7.4) μg/m^3^	Clinical Practice Research Datalink
Italy	11	Cesaroni et al. 2013^*e*^	12,208 (mortality)	1,265,058	2001–2010	Air dispersion	43.6 (8.4) μg/m^3^	Rome Longitudinal Study
Germany	12	Heinreich et al. 2013^*e*^	41 (mortality)	4,752	1980–2008	Fixed-site monitor	39 (NR) μg/m^3^	German Women’s Health Study
Denmark	NA	Raaschou-Nielsen et al. 2010^*f*^	679 (incidence)	4,160	1970–2001	Spatiotemporal model	37.6 (NR) μg/m^3^	Copenhagen City Heart Study; Copenhagen male study
Denmark	NA	Raaschou-Nielsen et al. 2011^*d,e*^	592 (incidence)	52,970	1993–2006	Spatiotemporal model	28.3 (NR) μg/m^3^	Danish Diet Cancer Health cohort
European Union	13	Raaschou-Nielsen et al. 2013^*d,e,f*^	2,095 (incidence)	312,944	1990s	Land use regression	NO_2_: 5.2 (2.5)–59.8 (10.6) μg/m^3^; NOx: 8.7 (5.7)–107.3 (24.3) μg/m^3^^*g*^	European Study of Cohorts for Air Pollution Effects (ESCAPE)
Asia
China	NA	Cao et al. 2011^*f*^	624 (mortality)	70,947	1991–2000	Fixed site monitor	50 (NR) μg/m^3^	China National Hypertension Epidemiology follow-up study
Japan	14	Katanoda et al. 2011	421 (mortality)	63,520	1983–1995	Fixed site monitor	1.2–33.7 ppb^*g*^	Three Prefecture Cohort Study
Japan	15	Yorifuji et al. 2013	116 (mortality)	13,412	1999–2009	Land use regression	22.0 (15.0) μg/m^3^	Shizuoka elderly cohort study
^***a***^Only studies of NO_2_ receive a study ID to indicate those studies that contributed to the subgroup analyses in Table 2; others are not applicable (NA). ^***b***^Studies using land use regression, spatiotemporal, inverse distance–weighted, or air dispersion models are more capable of addressing intra-city comparisons of air pollution exposure because they more closely estimate residential exposure to air pollutants. Each technique achieves this goal in a similar but distinct manner: Inverse distance weighting accounts for the distance from monitoring sites to the individual’s residence; land use regression includes relevant environmental GIS–derived variables in regression analyses; spatiotemporal models create air pollutant surfaces that vary by space and time using relevant environmental GIS–derived variables; and air dispersion models use relevant environmental GIS–derived variables, but assume that air pollutant transport follows a deterministic process based on Gaussian plume equations. In contrast, fixed-site monitors apply single exposure values obtained at monitoring sites to the populations surrounding the monitors, and are most useful for intercity comparisons. ^***c***^NR indicates that exposure means (± SDs) were not reported. Nafstad et al. (2003) report the median value of NO_x_. ^***d***^Studies that use a case–control design. Unless otherwise noted, all studies are cohort designs. ^***e***^Studies that examine lung cancer risk associated with distance to roadways or traffic density. These measures are explicitly described in Figure 2. ^***f***^Studies of NO_x_. All other studies examine NO_2_ exposure. ^***g***^We report the highest and lowest cohort specific mean (± SD) of measured air pollution from Raaschou-Nielsen et al. (2013) among the cohorts pooled for the ESCAPE study. Katanoda et al. (2011) report the range of observed NO_2_ values.								

**Figure 1:**
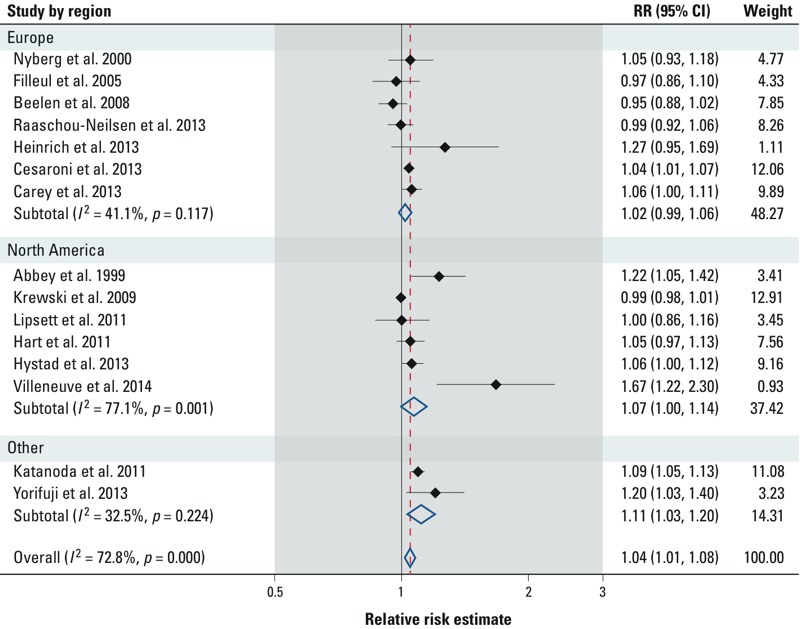
Forest plot of study-specific estimates of relative risk (RR) of lung cancer associated with a 10-μg/m^3^ increase in exposure to NO_2_. The meta-estimate and weights in the forest plot are estimated from random effects meta-analyses.

**Figure 2:**
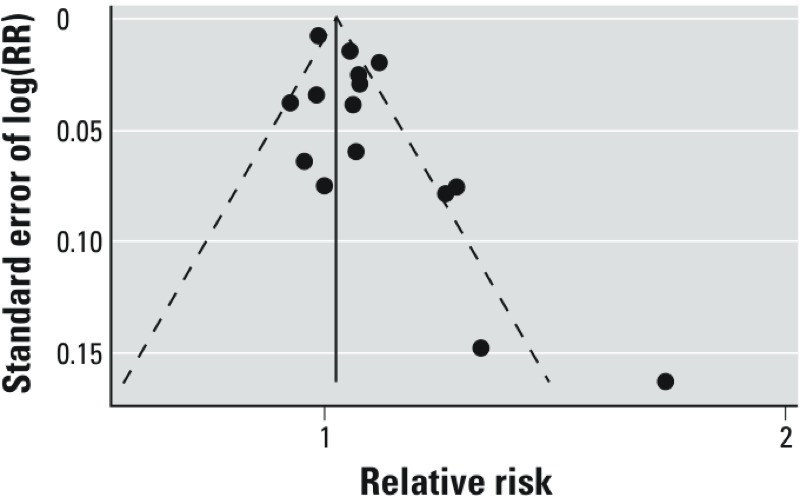
Funnel plot of study-specific estimates of the relative risk of lung cancer associated with a 10-μg/m^3^ increase in exposure to NO_2_. The meta-estimate represented by the vertical solid line of the funnel plot is based on a fixed-effects meta-analysis. Fixed-effects meta-analyses are required to assess the potential for publication bias, and assume that there is no between-study variation.

*NO_2_/NO_x_ and lung cancer*. Figure 1 and Table 2 summarize the studies examining the change in lung cancer incidence and mortality associated with traffic exposure as measured by NO_2_. The overall meta-estimate of the change in lung cancer incidence or mortality per 10-μg/m^3^ increase in exposure is 4% [95% confidence interval (CI): 1%, 8%], with an *I*^2^ estimate of 72.8%. Region-specific estimates vary; for Europe, North America, and Japan, the meta-estimates are 2% (95% CI: –1%, 6%), 7% (95% CI: 0%, 14%), and 11% (95% CI: 3%, 20%), respectively. The funnel plot is visually asymmetrical (Figure 2). Trim-and-fill analyses indicate that five hypothetical studies would need to have been observed to create a symmetrical funnel plot; the meta-estimate for the change in lung cancer per 10-μg/m^3^ increase in NO_2_ recalculated based on inclusion of these unobserved studies was attenuated toward the null: 1% (95% CI: –2%, 5%). A cohort study by Naess et al. (2007) of all residents in Oslo, Norway, who were 51–90 years of age met all inclusion criteria, but could not be included in our analyses because risk estimates from this study were based on quartiles of exposure. However, the results of that work support an increase in lung cancer risk associated with NO_2_; percent change in lung cancer mortality associated with a 1-quartile increase in NO_2_ for men and women age 51–70 years were 7% (95% CI: –3%, 18%) and 23% (95% CI: 10%, 38%), and for men and women age 71–90 years were 9% (95% CI: –2%, 20%) and 12% (95% CI: –2%, 27%), respectively (Naess et al. 2007).

**Table 2:** Meta-estimates of the association between lung cancer and a 10-μg/m^3^ increase in NO_2_.

Estimate	Meta-estimate (95% CI)	*I*^2^ (%)	Studies included (by ID)^*a*^
Full meta-estimate	1.04 (1.01, 1.08)	72.8	All
Continent
Europe	1.02 (0.99, 1.06)	41.1	7, 8–13
North America	1.07 (1.01, 1.14)	77.1	1–6
Asia	1.11 (1.03, 1.20)	32.5	14, 15
Exposure assessment method
Fixed-site monitor	1.05 (0.98, 1.13)	80.7	1, 2, 4, 8, 12, 14
Other	1.04 (1.00, 1.08)	60.5	3, 6, 7, 9–11, 13, 15
Confounder adjustment
Smoking status	1.04 (1.00, 1.08)	71.8	1, 2, 4, 5, 7, 8, 9, 12–15
Socioeconomic status/income	1.04 (0.98, 1.11)	68.8	4, 6, 7, 9, 11, 13, 15
Education	1.03 (1.00, 1.07)	68.2	1, 2, 5, 8, 10–13
Occupation	1.02 (0.99, 1.04)	40.9	2–5, 7, 8, 11, 13
^***a***^Study IDs are listed in Table 1.			

**Figure 3:**
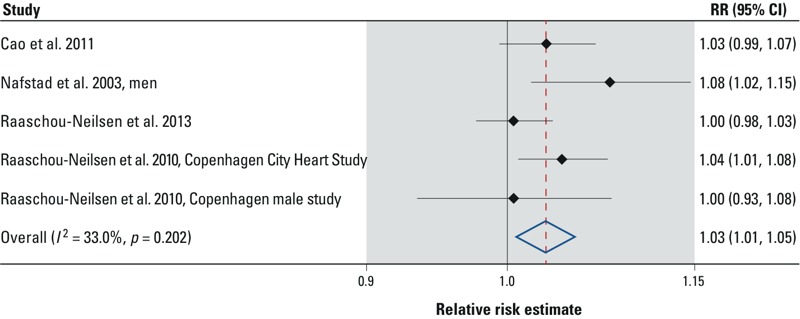
Forest plot of study specific estimates of relative risk (RR) of lung cancer associated with a 10-μg/m^3^ increase in exposure to NO_x_.

The meta-estimate for the change in lung cancer associated with a 10-μg/m^3^ increase in measured NO_x_ is 3% (95% CI: 1%, 5%), based on five relative risk estimates for lung cancer associated with a 10-μg/m^3^ increase in NO_x_ derived from four studies: 1.03 (0.99, 1.07) (Cao et al. 2011), 1.08 (1.02, 1.15) (Nafstad et al. 2003), 1.00 (0.98, 1.03) (Raaschou-Nielsen et al. 2013), and 1.04 (1.01, 1.08) and 1.00 (0.93, 1.08) (Raaschou-Nielsen et al. 2010). Figure 3 provides a visual summary of these study-specific relative risk estimates. The smaller number of risk estimates available does not allow for detailed analyses of sensitivity to confounder adjustment or other subgroup-specific analyses. However, it is notable that the meta-estimate for lung cancer associated with NO_x_ is similar to that of NO_2_, but slightly more precise.

Table 2 summarizes sensitivity and subgroup analyses for lung cancer associated with measured NO_2_. When restricted to studies that adjusted for confounding due to smoking status, socioeconomic status/income, education, or occupation, meta-estimates are largely unchanged; point estimates are similar, and confidence intervals largely overlap with that of the overall meta-estimate. Further, when we divide studies by the exposure assessment technique used, the meta-estimates are identical, or nearly identical, to the overall meta-estimate, but with slightly different confidence intervals. Influence analyses suggested that no single study influenced the overall meta-estimate (see Supplemental Material, Table S1).

**Figure 4:**
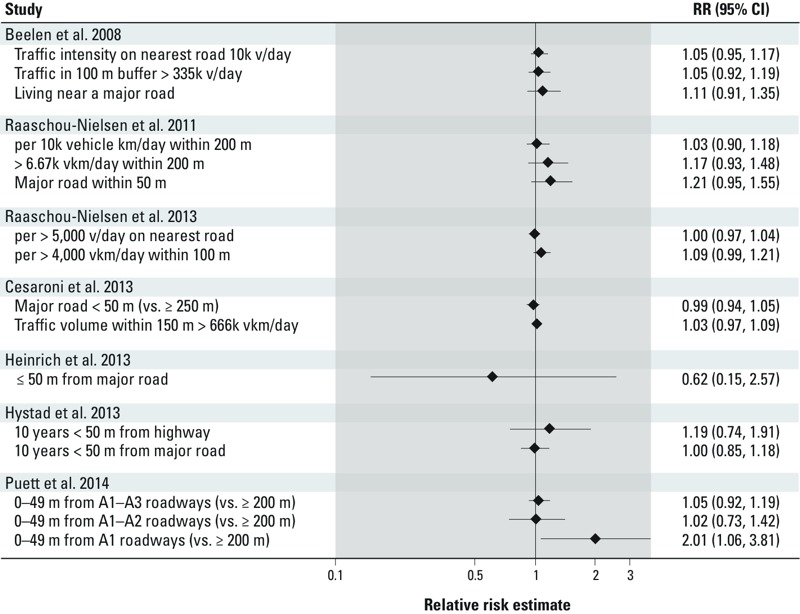
Relative risk of lung cancer associated with measures of traffic exposure. Abbreviations: k, 1,000; km, kilometers; m, meters; v, vehicles; vkm, vehicle kilometers.

Evidence from studies that consider distance from traffic sources or volume of traffic at roads nearest to residence is summarized in Figure 4. Each study uses a unique exposure contrast; thus, we do not provide a meta-estimate of these study specific measures of lung cancer associated with traffic exposure. We only note that many of the estimates reported are either close to or include a null association within the bounds of 95% confidence intervals reported.

## Discussion

We provide a systematic review and meta-analyses of the evidence regarding the association of NO_2_, NO_x_, and distance to roadways or volume of vehicles as markers of exposure to traffic-based air pollution. The studies we considered represent many regions of the world and vary by the level of exposure measured, exposure assessment technique, and potential confounders considered. The results of the overall meta-analyses support a relationship between lung cancer and exposure to air pollution from traffic sources.

Analyses stratified by continent suggest the possibility of geographic variability in the magnitude of the relative risk. However, confidence intervals largely overlap, which limits the ability to draw conclusions about heterogeneity. In addition, exposure lags varied in studies from 0 years to 20 years, but were not reported in some studies; Raaschou-Nielsen et al. (2010) found no difference in risk estimates when using a 0-year versus a 10-year exposure lag. Trim-and-fill analyses for NO_2_ meta-estimates indicated the potential for publication bias, which, if present, would shift results away from the null. To obtain the null result suggested by the trim-and-fill analyses, we would need to assess five hypothetical (i.e., unobserved) studies that obtained results that are the inverse of five studies with the largest and least precise risk estimates. Otherwise, the meta-estimate for NO_2_ was robust to confounders considered and methods of exposure assessment used by the authors of each study. No formal assessment of study quality was conducted as part of this meta-analysis.

Epidemiologic evidence alone does not allow us to assess nitrogen oxides as a causative agent in development of lung cancer. Nitrogen oxides are not thought to be carcinogenic agents (Valavanidis et al. 2008). Rather, they serve as a marker for other pollutants formed in high-temperature combustion of fossil fuels, a mixture that also includes fine particles. The role of nitrogen oxides as a marker for traffic-related air pollution is most plausible in urban settings, where traffic is often the primary source of NO_x_ in the atmosphere and the main source of variability in NO_x_ levels. NO_x_ is the sum of NO (nitrogen oxide) and NO_2_; because NO_2_ is a secondary oxidation product of atmospheric reactions involving NO, the latter of which is directly emitted in combustion, NO_x_ is considered a better indicator of traffic pollution than is NO_2_ alone. Because NO_2_ is a component of NO_x_ and will, therefore, always be present at a lower concentration, one would expect a lower effect estimate for NO_x_ (per 1-μg/m^3^) compared with that for NO_2_, assuming that either was the true carcinogenic agent in the traffic pollution mixture. Recent studies have shown that NO_x_ is highly correlated with combustion by-products, such as volatile organic compounds and carbonyls (Brook et al. 2007; Curren et al. 2006), many of which are known carcinogens. Combustion of fuel from motor vehicles can contribute a significant portion of outdoor air pollution, particularly in urban areas.

Because cancer is an outcome that is believed to develop over many years, exposure modeling that better approximates long-term, individual-level exposures may provide more valid estimates of the carcinogenic potential of traffic, or other, air pollution. This is distinct from studies of outcomes with sudden onset; the latter can rely on aggregate measures, such as fixed-site monitors and short-term exposure information, because these measures adequately describe relevant, short-term exposure episodes such as high traffic or temperature days (Gram et al. 2003). Even when modeling techniques are used, the residential-level exposures assigned to an individual are, at best, an approximation of individual-level exposure. Further, each modeling technique will vary in its ability to provide individual-level estimates of exposure to pollution. Land use regression, used in four studies considered here, can provide better prediction around major highways where traffic patterns are better known, but not in areas without dense monitoring systems (Jerrett et al. 2005). Alternately, air dispersion models can account for spatiotemporal differences in pollution concentrations, but rely on assumptions that dispersion patterns are Gaussian, which may not be valid (Bellander et al. 2001). Both of these techniques, as well as other spatiotemporal models, attempt to assess within-city contrasts that cannot be accounted for with fixed-site monitors. In short, all exposure-modeling techniques provide, at best, an approximation of individual exposure. A detailed comparison of the most commonly used exposure assessment techniques was provided by Jerrett et al. (2005). Results of our meta-analyses for NO_2_ were robust to stratification by exposure assessment technique; that is, results were nearly identical regardless of whether or not an exposure modeling technique was used in place of fixed site monitors. It is noteworthy that the meta-estimate restricted to exposure modeling techniques was slightly more precise than that obtained from studies using fixed-site monitors.

Similarly, risk estimates for distance to traffic or roadways, summarized in Figure 2, are crude in that they do not postulate what an individual’s exposure at a residence would be from traffic sources. Distance to roadways can be a marker of socioeconomic status, the influence of which can be difficult to fully take into account. Also, the exposure measures for which the relative risks are estimated are inconsistent across studies; thus, it is impossible to summarize them in any quantitatively meaningful way.

A recent meta-analysis (Hamra et al. 2014) showed a strong and consistent association between exposure to particulate matter and lung cancer; the results, like those presented here, were robust to different confounder sets considered, as well as variation in exposure assessment techniques and study locations (Hamra et al. 2014). In the present review we considered indicators of exposure to motor vehicle traffic (nitrogen oxides and distance). Vehicle traffic also generates particulate matter, which can also arise from numerous anthropogenic and natural processes outside of traffic. By considering markers of traffic exposure, we hope to provide a better understanding of the association of lung cancer with pollutant mixtures that are characteristic of urban and highly populated environments. The results for NO_x_ and NO_2_ are compatible with the hypothesis that traffic-related air pollution increases the risk of lung cancer. Further, the IARC has classified exposure to diesel exhaust as a Group I carcinogen (Benbrahim-Tallaa et al. 2012). The results of our meta-analyses are consistent with this previous determination, and provide further support for the carcinogenicity of air pollution.

## Supplemental Material

(91 KB) PDF

## References

[r1] Abbey DE, Nishino N, McDonnell WF, Burchette RJ, Knutsen SF, Beeson WL (1999). Long-term inhalable particles and other air pollutants related to mortality in nonsmokers.. Am J Respir Crit Care Med.

[r2] Beelen R, Hoek G, van den Brandt PA, Goldbohm RA, Fischer P, Schouten LJ (2008). Long-term exposure to traffic-related air pollution and lung cancer risk.. Epidemiology.

[r3] Beeson WL, Abbey DE, Knutsen SF (1998). Long-term concentrations of ambient air pollutants and incident lung cancer in California adults: results from the AHSMOG study. Adventist Health Study on Smog.. Environ Health Perspect.

[r4] Bellander T, Berglind N, Gustavsson P, Jonson T, Nyberg F, Pershagen G (2001). Using geographic information systems to assess individual historical exposure to air pollution from traffic and house heating in Stockholm.. Environ Health Perspect.

[r5] Benbrahim-Tallaa L, Baan RA, Grosse Y, Lauby-Secretan B, El Ghissassi F, Bouvard V (2012). Carcinogenicity of diesel-engine and gasoline-engine exhausts and some nitroarenes.. Lancet Oncol.

[r6] Brauer M, Hoek G, Van Vliet P, Meliefste K, Fischer PH, Wijga A (2002). Air pollution from traffic and the development of respiratory infections and asthmatic and allergic symptoms in children.. Am J Respir Crit Care Med.

[r7] Brook JR, Burnett RT, Dann TF, Cakmak S, Goldberg MS, Fan X (2007). Further interpretation of the acute effect of nitrogen dioxide observed in Canadian time-series studies.. J Expo Sci Environ Epidemiol.

[r8] Brunekreef B, Janssen NA, de Hartog J, Harssema H, Knape M, van Vliet P (1997). Air pollution from truck traffic and lung function in children living near motorways.. Epidemiology.

[r9] Brunekreef B Stewart AW Anderson HR Lai CK Strachan DP Pearce N 2009 Self-reported truck traffic on the street of residence and symptoms of asthma and allergic disease: a global relationship in ISAAC phase 3. Environ Health Perspect 117 1791 1798; 10.1289/ehp.0800467 20049134 PMC2801184

[r10] Cao J, Yang C, Li J, Chen R, Chen B, Gu D (2011). Association between long-term exposure to outdoor air pollution and mortality in China: a cohort study.. J Hazard Mater.

[r11] Carey IM, Atkinson RW, Kent AJ, van Staa T, Cook DG, Anderson HR (2013). Mortality associations with long-term exposure to outdoor air pollution in a national English cohort.. Am J Respir Crit Care Med.

[r12] Cesaroni G Badaloni C Gariazzo C Stafoggia M Sozzi R Davoli M 2013 Long-term exposure to urban air pollution and mortality in a cohort of more than a million adults in Rome. Environ Health Perspect 121 324 331; 10.1289/ehp.1205862 23308401 PMC3621202

[r13] Curren KC, Dann TF, Wang DK (2006). Ambient air 1,3-butadiene concentrations in Canada (1995–2003): seasonal, day of week variations, trends, and source influences.. Atmos Environ.

[r14] DerSimonian R, Laird N (1986). Meta-analysis in clinical trials.. Control Clin Trials.

[r15] Duval S, Tweedie R (2000). A nonparametric “trim and fill” method of accounting for publication bias in meta-analysis.. J Am Stat Assoc.

[r16] Filleul L, Rondeau V, Vandentorren S, Le Moual N, Cantagrel A, Annesi-Maesano I (2005). Twenty five year mortality and air pollution: results from the French PAARC survey.. Occup Environ Med.

[r17] Gram F, Nafstad P, Håheim LL (2003). Estimating residential air pollution exposure among citizens in Oslo 1974–1998 using a geographical information system.. J Environ Monit.

[r18] Hamra GB Guha N Cohen A Laden F Raaschou-Nielsen O Samet JM 2014 Outdoor particulate matter exposure and lung cancer: a systematic review and meta-analysis. Environ Health Perspect 122 906 911; 10.1289/ehp.1408092 24911630 PMC4154221

[r19] Hart JE, Garshick E, Dockery DW, Smith TJ, Ryan L, Laden F (2011). Long-term ambient multipollutant exposures and mortality.. Am J Respir Crit Care Med.

[r20] HEI (Health Effects Institute). (2010). Traffic-related air pollution: a critical review of the literature on emissions, exposure, and health effects. HEI Panel on the Health Effects of Traffic-Related Air Pollution. Special Report 17.

[r21] Heinrich J, Thiering E, Rzehak P, Krämer U, Hochadel M, Rauchfuss KM (2013). Long-term exposure to NO_2_ and PM_10_ and all-cause and cause-specific mortality in a prospective cohort of women.. Occup Environ Med.

[r22] Higgins JPT, Green S, eds. (2008). Cochrane Handbook for Systematic Reviews of Interventions.

[r23] Higgins JP, Thompson SG, Deeks JJ, Altman DG (2003). Measuring inconsistency in meta-analyses.. BMJ.

[r24] Hoek G, Brunekreef B, Goldbohm S, Fischer P, van den Brandt PA (2002). Association between mortality and indicators of traffic-related air pollution in the Netherlands: a cohort study.. Lancet.

[r25] Hystad P, Demers PA, Johnson KC, Carpiano RM, Brauer M (2013). Long-term residential exposure to air pollution and lung cancer risk.. Epidemiology.

[r26] Jerrett M, Arain A, Kanaroglou P, Beckerman B, Potoglou D, Sahsuvaroglu T (2005). A review and evaluation of intraurban air pollution exposure models.. J Expo Anal Environ Epidemiol.

[r27] Jerrett M, Burnett RT, Beckerman BS, Turner MC, Krewski D, Thurston G (2013). Spatial analysis of air pollution and mortality in California.. Am J Respir Crit Care Med.

[r28] Katanoda K, Sobue T, Satoh H, Tajima K, Suzuki T, Nakatsuka H (2011). An association between long-term exposure to ambient air pollution and mortality from lung cancer and respiratory diseases in Japan.. J Epidemiol.

[r29] Krewski D, Jerrett M, Burnett RT, Ma R, Hughes E, Shi Y (2009). Extended follow-up and spatial analysis of the American Cancer Society study linking particulate air pollution and mortality.. Res Rep Health Eff Inst.

[r30] Lipsett MJ, Ostro BD, Reynolds P, Goldberg D, Hertz A, Jerrett M (2011). Long-term exposure to air pollution and cardiorespiratory disease in the California Teachers Study cohort.. Am J Respir Crit Care Med.

[r31] Naess Ø, Nafstad P, Aamodt G, Claussen B, Rosland P (2007). Relation between concentration of air pollution and cause-specific mortality: four-year exposures to nitrogen dioxide and particulate matter pollutants in 470 neighborhoods in Oslo, Norway.. Am J Epidemiol.

[r32] Nafstad P, Håheim LL, Oftedal B, Gram F, Holme I, Hjermann I (2003). Lung cancer and air pollution: a 27 year follow up of 16 209 Norwegian men.. Thorax.

[r33] Nyberg F, Gustavsson P, Järup L, Bellander T, Berglind N, Jakobsson R (2000). Urban air pollution and lung cancer in Stockholm.. Epidemiology.

[r34] Puett RC Hart JE Yanosky JD Spiegelman D Wang M Fisher JA 2014 Particulate matter air pollution exposure, distance to road, and incident lung cancer in the Nurses’ Health Study cohort. Environ Health Perspect 122 926 932; 10.1289/ehp.1307490 24911062 PMC4154215

[r35] Raaschou-Nielsen O, Andersen ZJ, Beelen R, Samoli E, Stafoggia M, Weinmayr G (2013). Air pollution and lung cancer incidence in 17 European cohorts: prospective analyses from the European Study of Cohorts for Air Pollution Effects (ESCAPE).. Lancet Oncol.

[r36] Raaschou-Nielsen O Andersen ZJ Hvidberg M Jensen SS Ketzel M Sørensen M 2011 Lung cancer incidence and long-term exposure to air pollution from traffic. Environ Health Perspect 119 860 865; 10.1289/ehp.1002353 21227886 PMC3114823

[r37] Raaschou-Nielsen O, Bak H, Sørensen M, Jensen SS, Ketzel M, Hvidberg M (2010). Air pollution from traffic and risk for lung cancer in three Danish cohorts.. Cancer Epidemiol Biomarkers Prev.

[r38] Tonne C Melly S Mittleman M Coull B Goldberg R Schwartz J 2007 A case–control analysis of exposure to traffic and acute myocardial infarction. Environ Health Perspect 115 53 57; 10.1289/ehp.9587 17366819 PMC1797833

[r39] Valavanidis A, Fiotakis K, Vlachogianni T (2008). Airborne particulate matter and human health: toxicological assessment and importance of size and composition of particles for oxidative damage and carcinogenic mechanisms.. J Environ Sci Health C Environ Carcinog Ecotoxicol Rev.

[r40] Villeneuve PJ, Jerrett M, Brenner D, Su J, Chen H, McLaughlin JR (2014). A case-control study of long-term exposure to ambient volatile organic compounds and lung cancer in Toronto, Ontario, Canada.. Am J Epidemiol.

[r41] Vineis P, Hoek G, Krzyzanowski M, Vigna-Taglianti F, Veglia F, Airoldi L (2006). Air pollution and risk of lung cancer in a prospective study in Europe.. Int J Cancer.

[r42] Vrijheid M Martinez D Manzanares S Dadvand P Schembari A Rankin J 2011 Ambient air pollution and risk of congenital anomalies: a systematic review and meta-analysis. Environ Health Perspect 119 598 606; 10.1289/ehp.1002946 21131253 PMC3094408

[r43] Yorifuji T, Kashima S, Tsuda T, Ishikawa-Takata K, Ohta T, Tsuruta K (2013). Long-term exposure to traffic-related air pollution and the risk of death from hemorrhagic stroke and lung cancer in Shizuoka, Japan.. Sci Total Environ.

