# Supplementary material for: Lung Cancer and Exposure to Nitrogen Dioxide and Traffic: A Systematic Review and Meta-Analysis
Source: Environ Health Perspect. 2015 Apr 14;123(11):1107–12. doi: 10.1289/ehp.1408882 (PMC4629738; doi:10.1289/ehp.1408882)
Supplement: (91 KB) PDF [file ehp.1408882.s001.acco.pdf]

Environ Health Perspect
doi: 10.1289/ehp.1408882

Note to Readers: *EHP* strives to ensure that all journal content is accessible to all readers. However, some figures and Supplemental Material published in *EHP* articles may not conform to 508 standards due to the complexity of the information being presented. If you need assistance accessing journal content, please contact ehp508@niehs.nih.gov. Our staff will work with you to assess and meet your accessibility needs within 3 working days.

Supplemental Material

Lung Cancer and Exposure to Nitrogen Dioxide and Traffic: A Systematic Review and Meta-Analysis

Ghassan B. Hamra, Francine Laden, Aaron J. Cohen, Ole Raaschou-Nielsen, Michael Brauer, and Dana Loomis

Table of Contents

Table S1. Influence analyses examining the lung cancer incidence and mortality associated with a 10 μg/m^3 change in exposure to NO.

Table S1. Influence analyses examining the lung cancer incidence and mortality associated with a 10 μg/m^3 change in exposure to NO_2.

Study Excluded	Estimate	95% CI
None	1.04	(1.01, 1.08)
Abbey et al 1999	1.04	(1.01, 1.07)
Beelen et al. 2008	1.05	(1.02, 1.09)
Carey et al. 2013	1.04	(1.01, 1.08)
Cesaroni et al. 2013	1.05	(1.01, 1.09)
Filleul et al. 2005	1.05	(1.01, 1.08)
Hart et al. 2011	1.04	(1.01, 1.08)
Heinrich et al. 2013	1.04	(1.01, 1.08)
Hystad et al. 2013	1.04	(1.01, 1.08)
Katanoda et al. 2011	1.04	(1.01, 1.07)
Krewski et al. 2009	1.05	(1.02, 1.09)
Lipsett et al. 2011	1.05	(1.01, 1.08)
Nyberg et al. 2000	1.04	(1.01, 1.08)
Raaschou-Neilsen et al. 2013	1.05	(1.01, 1.09)
Villeneuve et al. 2013	1.04	(1.01, 1.07)
Yorifuji et al. 2013	1.04	(1.01, 1.07)
